# An IFIH1 gene polymorphism associated with risk for autoimmunity regulates canonical antiviral defence pathways in Coxsackievirus infected human pancreatic islets

**DOI:** 10.1038/srep39378

**Published:** 2016-12-21

**Authors:** Erna Domsgen, Katharina Lind, Lingjia Kong, Michael H. Hühn, Omid Rasool, Frank van Kuppeveld, Olle Korsgren, Riitta Lahesmaa, Malin Flodström-Tullberg

**Affiliations:** 1 The Center for Infectious Medicine, Department of Medicine HS, Karolinska Institutet, Karolinska University Hospital, Stockholm, 141 86, Sweden; 2 Turku Centre for Biotechnology, University of Turku and Åbo Akademi University, Turku, 205 20, Finland; 3 Virology Division, Department of Infectious Diseases and Immunology, Faculty of Veterinary Medicine, Utrecht University, Utrecht, 3584, The Netherlands; 4 Department of Immunology, Genetics, and Pathology, Rudbeck Laboratory, Uppsala University, Uppsala, 751 05, Sweden; 5 Institute of Biosciences and Medical Technologies, University of Tampere, Tampere, 33520, Finland

## Abstract

The IFIH1 gene encodes the pattern recognition receptor MDA5. A common polymorphism in IFIH1 (rs1990760, A946T) confers increased risk for autoimmune disease, including type 1-diabetes (T1D). Coxsackievirus infections are linked to T1D and cause beta-cell damage *in vitro*. Here we demonstrate that the rs1990760 polymorphism regulates the interferon (IFN) signature expressed by human pancreatic islets following Coxsackievirus infection. A strong IFN signature was associated with high expression of IFNλ1 and IFNλ2, linking rs1990760 to the expression of type III IFNs. In the high-responding genotype, IRF-1 expression correlated with that of type III IFN, suggesting a positive-feedback on type III IFN transcription. In summary, our study uncovers an influence of rs1990760 on the canonical effector function of MDA5 in response to an acute infection of primary human parenchymal cells with a clinically relevant virus linked to human T1D. It also highlights a previously unrecognized connection between the rs1990760 polymorphism and the expression level of type III IFNs.

The interferon-induced helicase-1 (IFIH1) gene encodes the melanoma differentiation associated protein 5 (MDA5), an intracellular pattern recognition receptor (PRR) of importance for the recognition of certain viruses. The MDA5 protein consists of two N-terminal caspase recruitment domains (CARDs), a central helicase domain, and a C-terminal domain (CTD). MDA5 monomers bind to long double stranded (ds)RNA molecules formed during the viral replication cycle. This initiates the formation of a MDA5 filament along the dsRNA, where multiple CARD domains assemble and recruit the signalling adaptor interferon promoter stimulator 1 (IPS1) (also denoted mitochondrial antiviral-signalling protein, MAVS). Activated IPS1/MAVS initiates the downstream signalling events leading to the activation of the transcription factors NF-κB and IRF-3, followed by the production of type I and III interferons (IFN)^1^,^2^,^3^,^4^.

The rapid production of IFNs is an essential component of the immune response against many viruses. Type I and III IFNs have potent antiviral activities; they act in an auto- and paracrine manner to induce the expression of interferon-stimulated genes (ISGs) leading to an antiviral state in uninfected cells. They also promote innate and adaptive immune responses^1^,^2^,^3^,^4^,^5^. Activation of type I and III IFN gene transcription is dependent on the activation of specific transcription factors, NF-κB and IRF-3. Besides these two, the transcription factor IRF-7 is required for the transcriptional activation of IFNα and possibly also IFN-λ2 and -3 genes. IRF-7 is constitutively expressed by some immune cells, but rarely by parenchymal cells. IRF-7 is often induced following exposure to JAK/STAT-activating cytokines, such as the IFNs themselves, thus establishing a positive feedback loop for IFN expression. IRF-1 is another IFN inducible transcription factor. It has binding sites in the type I and III IFN promoters and can also contribute to a positive feedback on IFN gene transcription. Recent studies have suggested that IRF-1 has a role in activating type III and not type I IFNs during infections with RNA viruses^3^,^6^,^7^.

The human IFIH1 gene contains numerous single nucleotide polymorphisms (SNPs). The non-synonymous SNP (nsSNP), rs1990760 (A946T), is associated with an increased risk for development of several autoimmune diseases including T1D diabetes, systemic lupus erythematosus and multiple sclerosis (e.g.^8^,^9^,^10^). Little is known on how this nsSNP regulates risk for development of these inflammatory diseases, and therefore information on the biological effects of this nsSNP may provide useful insights.

Previous studies using ectopic overexpression of MDA5 in cell lines have reached contrasting conclusions with regards to the impact of the rs1990760 nsSNP on the activities of MDA5. Some studies reported no influence on function^11^,^12^,^13^, while another study suggested that the risk allele T (946 T) encodes a constitutively active MDA5 protein that is unable to respond to a natural ligand (encephalomyocarditis virus, EMCV^14^). These contrasting results illustrate that additional studies are required to reveal if and how the rs1990760 affects the function of MDA5.

Recent studies have shown that the functional impact of disease-associated SNPs may vary depending on the cell type studied (e.g.^15^). This, together with the recent notion that the antiviral effector mechanisms of MDA5 may vary in response to different types of viruses^14^,^16^,^17^, suggest that studies using relevant human primary cells and pertinent natural ligands (i.e. clinically relevant viruses rather than viral mimics such as poly I:C) are needed to gain a better understanding of the function of the disease-associated variants of IFIH1.

Enterovirus infections, in particularly those with Coxsackievirus (CVBs), have been associated with the development of T1D in humans. The link between CVB infections and T1D is supported by case reports, epidemiological data and by observations indicating that enteroviruses are present in the remaining beta cells of individuals with T1D diabetes^18^,^19^,^20^,^21^,^22^,^23^,^24^,^25^. We have shown that human pancreatic islets express MDA5^26^, and together with others demonstrated that MDA5 is important in the host immune response to CVBs^27^,^28^. Human pancreatic islets are easily and reproducibly infected by CVBs *in vitro* (e.g.^25^,^26^,^29^). This makes the islets a relevant and useful model to study a primary cell response to a CVB infection and to assess whether the rs1990760 nsSNP affect this response. In the present study, we used human pancreatic islets (primary cells) and a virus of clinical relevance (Coxsackievirus) as natural ligand, to provide a better understanding of the functional consequences of this polymorphism. Our study reveals previously not described relationships between the rs1990760 polymorphism and the magnitude of the innate response to Coxsackievirus infection.

## Results

### Whole transcriptome analysis identifies an IFN signature in CVB3 infected islets

**Figure 1:**
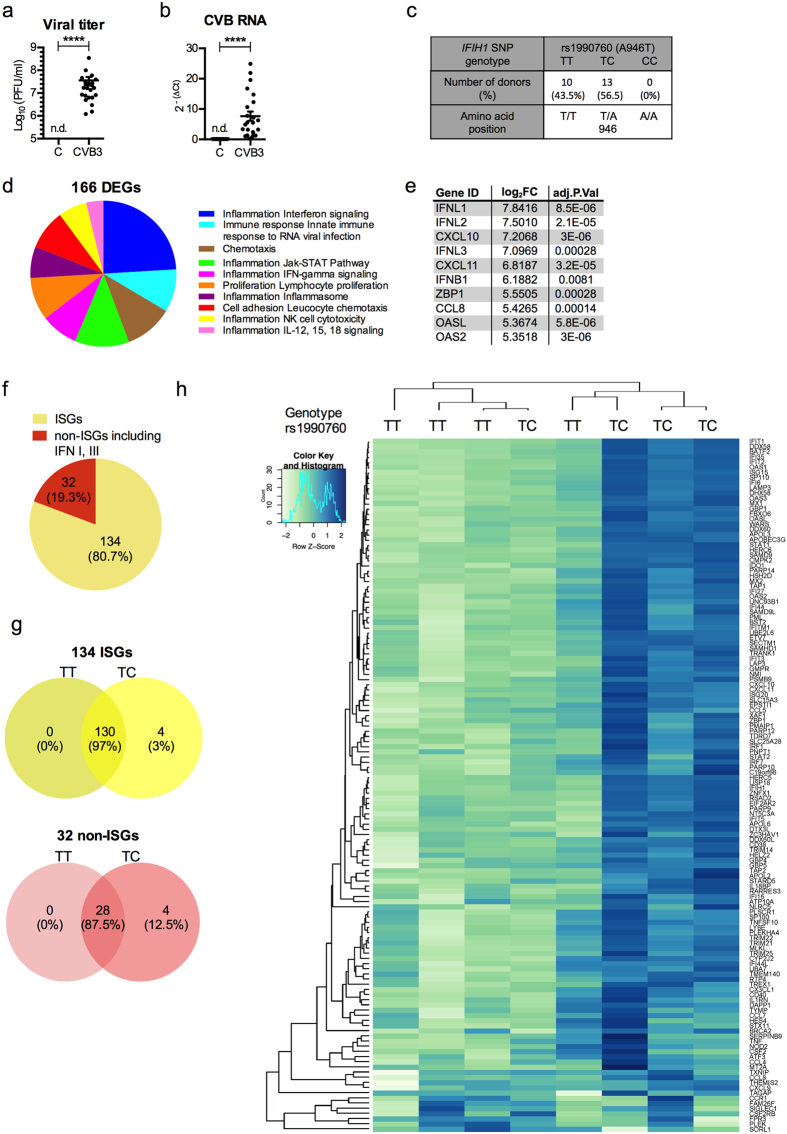
RNAseq analysis identifies differences in gene expression between CVB3 infected pancreatic islets carrying the rs1990760 TT and TC genotypes. (**a** and **b**) Human islets from 23 donors were infected in mock or with CVB3. Islets and supernatants were collected at 48 h p.i. The titers of replicating virus particles in supernatants from infected islets (**a**) were measured by a standard plaque assay and presented as Log_10_ (PFU/ml). The expression levels of virus RNA (**b**) in CVB3 infected islets were measured using quantitative real-time PCR. The expression levels were normalized to that of GAPDH and presented as 2^−(ΔCt)^ (relative expression). Data is shown as mean ± SEM, ****p < 0.0001, Wilcoxons matched-pairs signed rank test. n.d., not detected. (**c**) Human islet donors included in the study were genotyped for the polymorphisms rs1990760 (A946T). Allele distribution is shown in numbers and as percentage of the whole cohort (n = 23). (**d**) Pie chart showing the enrichment of process networks based on the 166 DEGs identified by RNAseq. Data was generated using the MetaCore^TM^ enrichment analysis tool with a cut off p-value of 0.05. The content of the process networks is defined and annotated by Thomson Reuters scientists. Each process represents a pre-set network of interactions characteristic for the process. (**e**) List of 10 genes displaying the highest log_2_ fold change in expression in infected human islets versus uninfected, as identified by RNAseq. (**f**) Pie chart showing the number and percentage of the 166 DEGs identified as ISGs or non-ISGs based on the Interferome database. (**g**) Venn diagrams showing the number of ISGs (upper diagram, yellow) and non-ISGs (including IFNs, lower diagram, red) commonly or differentially expressed in the two rs1990760 genotype populations (TT and TCs). (**h**) Heat map of differentially expressed ISGs (n = 134). Log_2_ (CPM) values were mean-centred and scaled for each gene (white, low expression difference; blue high expression difference). The Pearson correlation was used to compute distances between genes and samples, and the clustering was performed using average linkage. Each column corresponds to one islet donor with indicated rs1990760 genotype and each row corresponds to a specific gene.

Infections with CVBs are associated with human diseases, including aseptic meningitis, myocarditis and autoimmune T1D. The serotype B3 (CVB3) replicates in human pancreatic islets, with a peak around 48–72 h post infection (p.i.) (26,29, data not shown). Moreover, robust changes in gene transcription can be measured at 48 h p. i. (29; Supplementary Fig. 1). Here, pancreatic islets from a total of 23 human donors were infected with CVB3 for 48 h. The islets were highly permissive to infection as shown by the measurements of high titers of infectious virus particles released into the tissue culture media (Fig. 1a) and concomitant expression of viral RNA in the infected islets (Fig. 1b).

Genotyping for rs1990760 was performed on DNA isolated from each individual islet batch. This analysis demonstrated that the islet cohort consisted of a mixture of donors carrying either the homozygous T1D diabetes risk genotype, TT, or TC, a genotype associated with protection from disease^10^. None of the donors carried the protective genotype CC (Fig. 1c). In parallel, the islet cohort was genotyped for the rs3747517 SNP (Supplementary Table S1). The results from this genotyping were in line with the previously published strong linkage disequilibrium with rs1990760^9^,^16^,^30^,^31^.

To address whether the rs1990760 nsSNP has an influence on the human islet response to CVB3 infection, islets from eight of the 23 donors (TT, n = 4; TC, n = 4) were subjected to whole-transcriptome analysis (RNAseq). Using a stringent statistical method (see Methods) this analysis identified a total of 166 mRNA transcripts that were significantly altered in infected compared to uninfected islets (FDR < 0.05, Fig. 1d, Supplementary Table S2). Functional annotation of the differentially expressed coding genes (DEGs) using MetaCore^TM^ (Thomson Reuters) suggested significant enrichment of biological pathways related to inflammation, innate immunity and response to viral infection (Fig. 1d). With the same software, we identified several transcription factors critical for robust antiviral responses (e.g. IRF-1, STAT-1 and -2), as well as both type I (IFNβ) and III IFN (IFNλ1–3) genes (Fig. 1e, and Supplementary Table S2). We also observed an increased expression of genes encoding negative regulators of IFN expression and signalling (e.g. USBP18, TRIM21, GBP4, Supplementary Table S2)^5^. Because we noted an upregulated expression of type I and III IFNs, which are known to induce a wide range of genes (so-called ISGs), we used the Interferome v2.0 database^32^ to identify how many of the DEGs were ISGs. This analysis revealed that the majority (80.7%) of the 166 DEGs were ISGs (Fig. 1f).

### The magnitude of the human islet immune response to CVB3 infection is linked to the rs1990760 genotype

Most of the DEGs identified in the RNAseq analysis (97%) were shared between donors carrying the TT and TC genotypes, with only 4 DEGs unique to donors with the TC genotype (Fig. 1g). Hierarchical clustering based on the 134 genes identified as ISGs distinguished two major clusters, in which the majority (3/4) of donors carrying the TT genotype were placed in a cluster with low relative gene expression levels. The majority of islets carrying the TC genotype (3/4) were clustered together based on high relative gene expression (Fig. 1h). Taken together, the transcriptome analysis demonstrated that CVB3 infection induces a robust immune response in human pancreatic islets. These studies also indicated that the strength of this response might be regulated by the rs1990760 genotype.

### The rs1990760 TC genotype is associated with a high type III IFN response and a strong IFN signature

**Figure 2:**
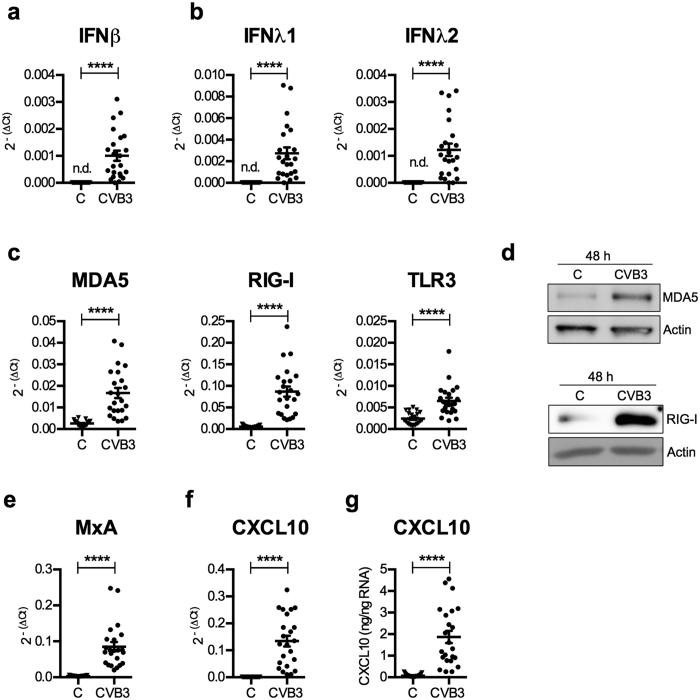
CVB3 infection of human pancreatic islets results in the upregulated expression of type I and III IFNs and ISGs. (**a–c,e** and **f**) mRNA expression levels of IFNβ (**a**), IFNλ1 and IFNλ2 (**b**), PRRs MDA5, RIG-I and TLR3 (**c**), MxA (**e**) and CXCL10 (**f**) were measured at 48 h p.i. in the mock- or CVB3 infected islets described in Fig. 1 (n = 23) using quantitative real-time PCR. The expression levels were normalized to that of GAPDH and presented as 2^−(ΔCt)^ (relative expression) for all islets. (**d**) Protein was isolated from a total of 6 islet donors. At 48 h p.i. the protein expression levels of MDA5 (3 donors) and RIG-I (3 donors) were measured in mock and CVB3 infected islets using western blot. Actin was used as loading control. Data from one donor is shown for each protein. The displayed figures are cropped images. Images of uncropped blots can be viewed in Supplementary Fig. S2. (**g**) The amount of secreted CXCL10 protein was measured in supernatant harvested from mock-infected and CVB3 infected islets (n = 23) using ELISA and expressed as ng/ng RNA. Data is shown as mean ± SEM, ****p < 0.0001, Wilcoxons matched-pairs signed rank test. n.d., not detected.

We next validated some of the results from the transcriptome analysis using conventional qPCR. By analysing cDNA from the whole islet cohort (n = 23) we verified that type I (IFNβ) and III IFNs (IFNλ1 and IFNλ) showed increased expression following CVB3 infection (Fig. 2a and b). Similarly, the expression levels of a number of the identified DEGs including PRRs (e.g. the IFIH1/MDA5 gene itself and DDX58/RIG-I), and genes encoding proteins involved in cellular antiviral defence (e.g. Mx1/MXA) or recruitment of lymphocytes and with relevance for T1D (CXCL10^33^) (Fig. 2c,e,f) were elevated. Western blot or ELISA measurements revealed that the induced gene expression translated into increased protein expression (Fig. 2d) and secretion (Fig. 2g).

We attempted to measure secreted type I and III IFNs, but our pilot studies using an ELISA and a sensitive bioassay failed to detect IFNs in the culture media of both control and CVB3 infected islets (data not shown). This is likely due to that enteroviruses, including CVB3, have developed mechanisms to impair type I and III IFN production and release^34^,^35^, and that the secreted IFNs are locally consumed due to binding to receptors expressed by the cultured cells.

**Figure 3:**
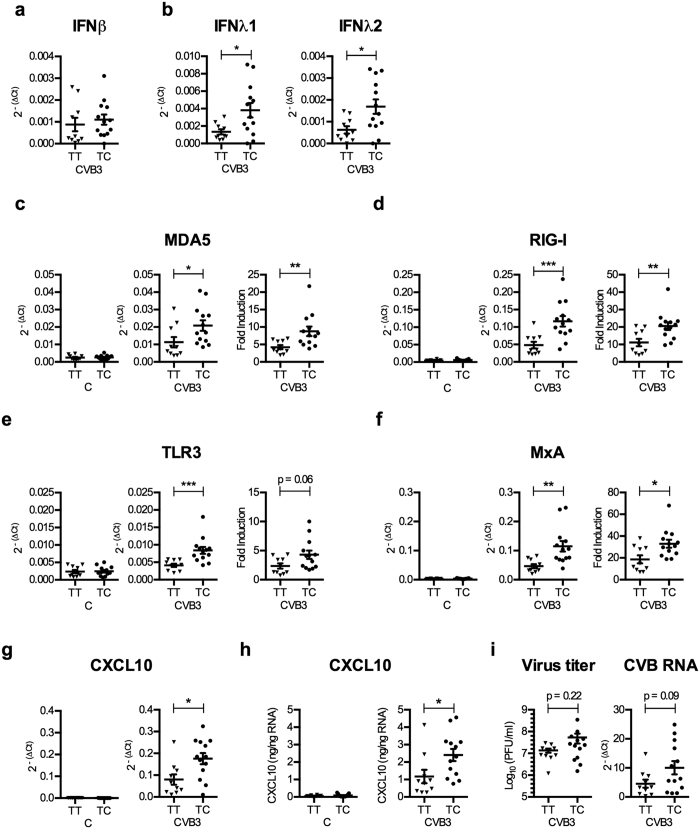
Homozygosity for the T (Thr946) allele of the rs1990760 polymorphism is associated with low human islet type III IFN and ISG mRNA expression levels, as well as amounts of secreted CXCL10, following CVB3 infection. (**a** and **b**) IFNβ (**a**), IFNλ1 and IFNλ2 (**b**) mRNA expression levels measured at 48 h p.i. in the CVB3 infected islets described in Fig. 2 (n = 23) were re-analysed and the donors stratified according to the rs1990760 genotype (TT or TC). The expression levels were normalized to that of GAPDH and presented as 2^−(ΔCt)^ (relative expression) for all islets. (**c–g**) mRNA expression levels of PRRs (MDA5, RIG-I, TLR3; (**c–e**), genes involved in antiviral defence (MxA; (**f**) or chemoattraction (CXCL10; (**g**), measured at 48 h p.i. in the mock-infected (left column, C) and CVB3 infected (two right columns, CVB3) islets described in Fig. 2 (n = 23) were re-analysed by dividing the donors according to the rs1990760 genotype (TT or TC). The expression levels were normalized to that of GAPDH and presented as 2^−(ΔCt)^ (relative expression; the two left columns), or normalized to GAPDH and compared to the expression levels in mock-infected islets from the same donor and presented as 2^−(ΔΔCt)^ (fold induction: the right column). Fold induction calculation was not applicable to IFNβ, IFNλ1–2 and CXCL10 mRNA, which were undetectable in mock-infected islets). (**h**) The amount of secreted CXCL10 protein (see Fig. 2g) was measured in supernatant harvested from mock-infected (left column, C) and CVB3 infected (right column, CVB3) islets using ELISA and expressed as ng/ng isolated RNA. The islet donors were stratified according to rs1990760 genotype TT or TC. (**i**) The data described in Fig. 2 was re-analysed and the islets were divided according to the rs1990760 genotype (TT or TC). Titers of replicating virus in supernatants harvested at 48 h p.i. are presented as Log_10_(PFU/ml). The expression levels of virus RNA in CVB3 infected islets were measured using quantitative real-time PCR. The expression levels were normalized to that of GAPDH and presented as 2^−(ΔCt)^ (relative expression). Data is shown as mean ± SEM, *p < 0.05, **p < 0.01, ***p < 0.001, Mann-Whitney test.

When stratifying our donors according to the rs1990760 genotype no statistically significant difference was detected in the expression of IFNβ mRNA between the islets carrying the TT and TC genotypes (Fig. 3a). In contrast, islets from donors carrying the TC genotype expressed significantly higher levels of type III IFNs when compared to islets carrying the TT genotype (p < 0.05; Fig. 3b). Similarly, TC islets expressed higher levels of the studied genes (ISGs), and the difference was observed also when comparing the fold induction (i.e. ΔΔCt-values) (p < 0.05 and p < 0.01; Fig. 3c–g). In line with the gene expression data, the secretion of the chemokine CXCL10 was also significantly higher in islets from donors with the TC genotype compared to those with the TT genotype (Fig. 3h). In contrast, the expression levels of the studied genes did not differ between the mock-infected islets of the TT and TC genotypes Fig. 3c–g). The titers of virus accumulating in the media during the whole 48 h culture period, as well as the expression of viral RNA at 48 h p.i., were not statistically different between the donors, although the latter was close to reaching significance (p = 0.09, Fig. 3i). Collectively, this shows that the human islets carrying the rs1990760 TC genotype have a higher type III IFN and ISG response to CVB3 than islets carrying the rs1990760 TT (risk) genotype.

### A high type III IFN response to CVB3 infection in TC donors is associated with a strong expression of IRF-1

**Figure 4:**
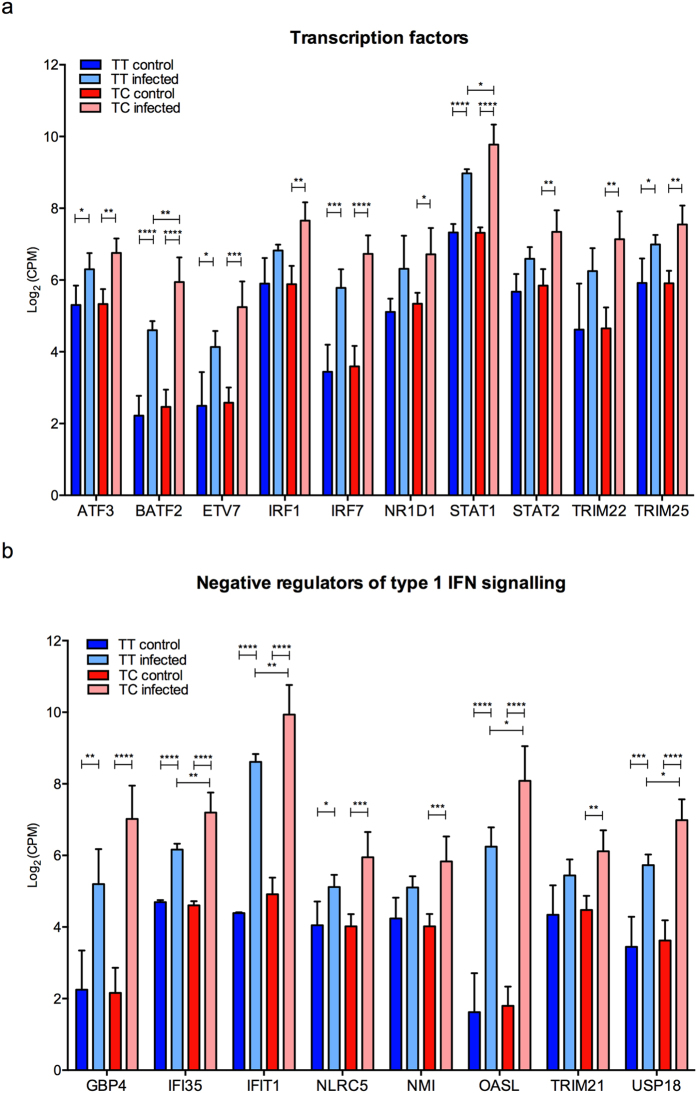
The gene expression of several transcription factors and genes involved in negative regulation of type 1 IFN signalling is increased in human islets upon CVB3 infection. RNA sequencing was performed on RNA isolated from non-infected and CVB3 infected primary human islets harvested at 48 h p.i. (8 donors in total; 4 TT and 4 TC) Among the genes demonstrating a differential expression between control and infected islets, 10 were identified as transcription factors and 8 have been described as negative regulators of type I IFN signalling^5^. The mRNA abundance is shown as log_2_(CPM) of identified transcription factors (**a**) and regulators of IFN signalling (**b**) from control and CVB3 infected islets carrying the rs1990760 TT (TT control, TT infected) or TC genotype (TC control, TC infected). Data is shown as mean ± SEM, *p < 0.05, **p < 0.01, ***p < 0.001, ****p < 0.0001, one-way ANOVA with Bonferroni correction.

IFN expression is regulated by the activities of transcription factors as well as negative regulators^3^,^36^,^37^. Our transcriptome analyses identified several transcription factors as being inducible by CVB3 infection (Fig. 4a). A few of these showed significantly different expression levels between the TT and TC genotypes (BATF2 and STAT1) (Fig. 4a). In addition to this, the expression of IRF-1 was significantly increased only in infected islets with the TC genotype (p < 0.01; Fig. 4a). Four negative regulators of type 1 IFN signalling, IFI35, IFIT1, OASL, USP18^5^, showed a different level of expression in the two genotypes (Fig. 4b). All four were expressed at a higher level in the TC genotype (high responders) as compared to the TT genotype (low responders). Thus, it is unlikely that these genes contributed to a higher expression of type III IFNs in islets from donors with the TC genotype per se, and these four genes were therefore not further studied.

**Figure 5:**
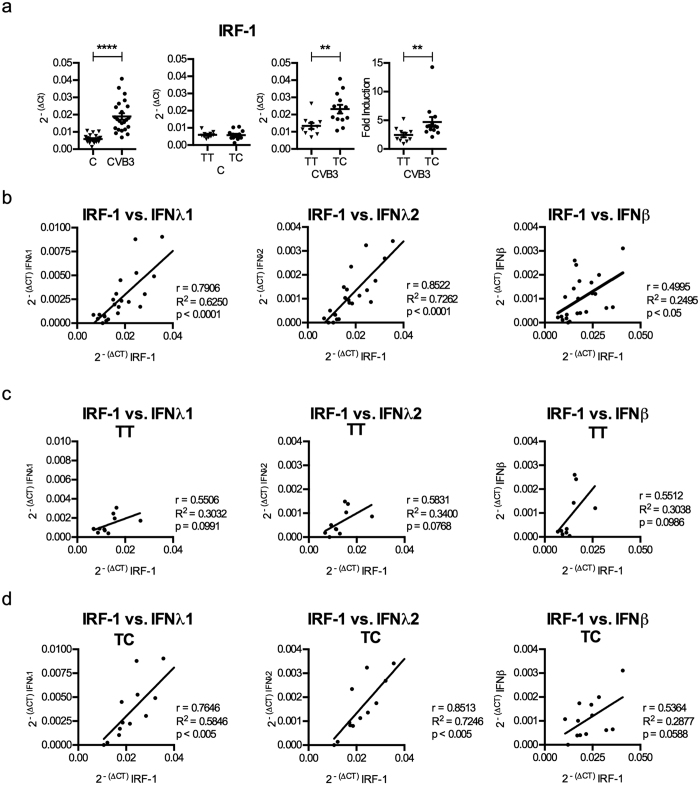
Higher expression levels of type III IFNs in TC donors correlate with the expression levels of the transcription factor IRF-1. (**a**) mRNA expression levels of IRF-1 were measured at 48 h p.i. in the mock- and CVB3 infected islets described in Fig. 1 (n = 23) using quantitative real-time PCR. The expression levels were normalized to that of GAPDH and presented as 2^−(ΔCt)^ (relative expression) for the whole islet cohort (first column). The results were re-analysed in that the donors were divided according to their rs1990760 genotype (TT or TC). The expression levels in the mock infected (C, second column) and CVB3 infected human islets (CVB3, third column) were normalized to that of GAPDH and presented as 2^−(ΔCt)^ (relative expression), or normalized to GAPDH and compared to the expression levels in mock-infected islets from the same donor and presented as 2^−(ΔΔCt)^ (fold induction: fourth column). (**b**) Correlation analysis was performed by comparing the mRNA levels for selected genes expressed by CVB3 infected islets at 48 h p.i. (n = 23). The 2^−(ΔCt)^ values of IRF-1 were correlated with that of IFNλ1, IFNλ2 and IFNβ in CVB3 infected islets. (**c,d**) The correlations described in (**b**) were separately performed for donors with the rs1990760 TT (**c**) and TC (**d**) genotypes. Linear regression and the correlation coefficient r, R^2^ and p values were calculated using Pearson correlation test (**b–d**). p < 0.05 was considered statistically significant.

The transcription factor IRF-1 has been shown to specifically regulate type III and not type I IFN expression during RNA virus infections^3^,^37^. Thus, we next analysed the expression of IRF-1 in the whole islet cohort by real-time PCR and confirmed that CVB3 infection induces the expression of IRF-1 (Fig. 5a). We found no difference in the basal expression levels of IRF-1 when control islets from TT and TC donors were compared (Fig. 5a). After CVB3 infection, islets with the TC genotype expressed higher levels of IRF-1 mRNA compared to islets with the TT genotype (Fig. 5a). Infected islets with the TC genotype also had a stronger increase in their IRF-1 mRNA expression relative to uninfected control islets (i.e. “fold induction”) as compared to islets with the TT genotype (Fig. 5a).

**Figure 6:**
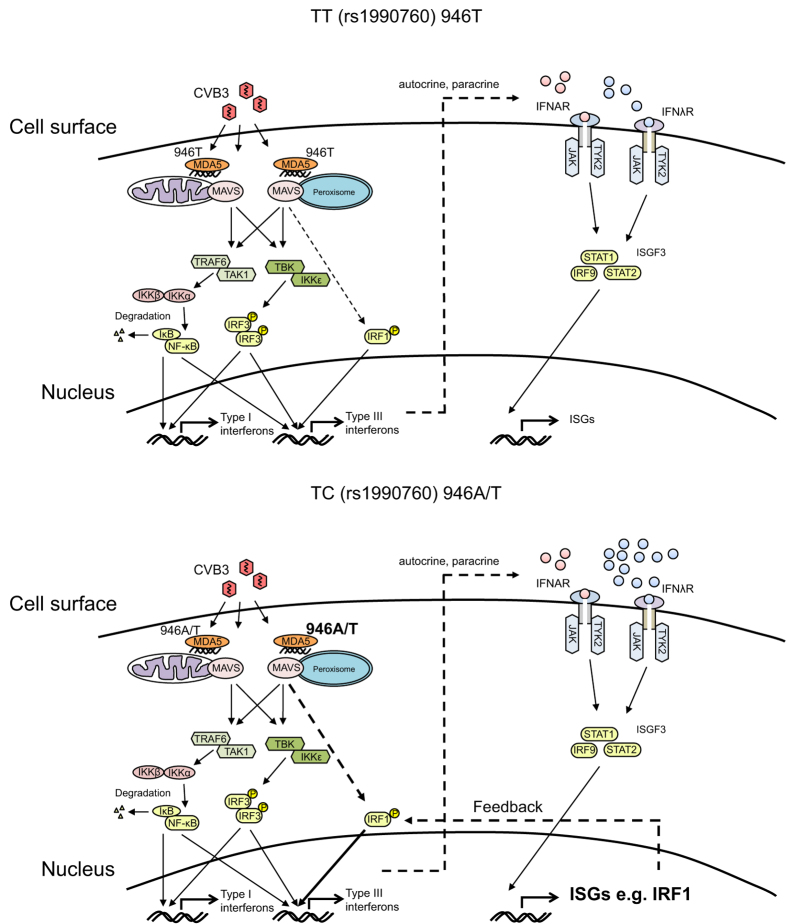
Proposed model of type I and type III IFN induction and action in islets from donors carrying the TT and TC rs1990760 genotypes. CVB3 infection of islets from donors carrying the rs1990760 TT genotype (MDA5, 946 T) (upper model) results in replication of viral RNA and the generation of a viral intermediate (dsRNA). MDA5 binds dsRNA and activates mitochondrial and peroxisomal associated MAVS. This initiates a signalling cascade leading to the phosphorylation and dimerization of IRF-3 through TBK-IKKε, and the degradation of IκB from the NF-κB complex through TRAF6 and TAK1. The activated transcription factors IRF-3 and NF-κB translocate to the nucleus where they bind to the promoter regions of type I and type III IFNs and initiate their expression. In addition, peroxisome associated MAVS leads through unknown mechanisms to the activation of the transcription factor IRF-1, which binds solely to the promoter regions of type III IFNs and selectively initiate their expression. Expressed and secreted type I and type III IFNs bind in an auto- and paracrine manner to their receptors IFNAR and IFNλR, respectively. This in turn activates JAK-TYK2 kinases leading to the formation of the ISGF3 complex and the expression of a large number of ISGs. Infection of islets from donors carrying the rs1990760 TC genotype (MDA5, 946 T/A) (lower model) results in stronger type III IFN gene expression compared to that induced in islets from TT donors, possibly through the peroxisome associated MAVS mediated activation of IRF-1. The stronger type III IFN expression is accompanied by a more robust ISG expression in TC donors compared to TT donors. This includes the expression of IRF-1, which may further amplify the type III IFN expression through a positive feedback mechanism. Why MDA5 activation in donors carrying the rs1990760 TC genotype leads to a stronger activation of peroxisome associated MAVS compared to mitochondrial associated MAVS is still to be discovered.

We next investigated whether there was a correlation between the levels of IRF-1 and type I and III IFN mRNA expression. When analysed for the entire human islet cohort the levels of expressed IRF-1 mRNA correlated with the expressed levels of IFNβ, IFNλ1 and IFNλ2 (Fig. 5b). However, when analysing the TT and TC donors separately it was found that the levels of IRF-1 mRNA correlated with the levels of the type III IFNs (IFNλ1 and IFNλ2) only in TC and not TT donors (Fig. 5c,d). This suggests that higher IRF-1 expression in the TC donors contributes to the stronger expression of type III IFNs via a feedback mechanism (Fig. 6).

### The expression of T1D-related genes does not differ between donors with the rs1990760 TT and TC genotypes

Risk for T1D development has been linked to polymorphisms in numerous genes besides IFIH1. Many of these genes are expressed by pancreatic beta cells and/or islets^38^. Using our gene transcription data (Supplementary Table S1) and the T1Dbase.org we identified some of these genes as having an altered expression level following CVB3 infection, including the upregulated expression of TAGAP, CD69 and IFIH1 itself, and down regulated expression of COBL (Supplementary Figure S3). It was however only the expression of the IFIH1 gene that showed a different expression level between donors with the TT and TC genotypes.

## Discussion

The present study uncovers a connection between the rs1990760 polymorphism and the magnitude of the innate immune response to a virus associated with human disease. To our knowledge, this is the first time that the rs1990760 polymorphism has been linked to the gene expression signature in primary human parenchymal cells infected with a clinically relevant virus.

The rs1990760 (A946T) nsSNP shows an association to numerous autoimmune diseases (e.g.^8^,^9^,^10^). The minor C allele (in Caucasians) is conserved among vertebrates and confers lower risk to develop autoimmunity. It was previously thought that the change from an alanine to a threonine in the 946^th^ amino acid position would not have an impact on MDA5’s functions^10^. Recent studies using high-resolution HDX-MS have however suggested that the amino acids 940–959 are involved in inter-molecular interactions^39^. Moreover, the CTD (amino acids 900–1025) has been shown to exhibit an auto-repressive function in the resting state^11^,^40^. These findings suggest that the amino acid substitution A946T might have an effect on MDA5 filament formation as well as the regulatory function of the CTD, both of which could affect protein function and down-stream antiviral effects. Here, we assessed whether the rs1990760 polymorphism affects the immune response to Coxsackievirus infection, and made a number of observations that oppose the early assumptions that this nsSNP would not have an impact on MDA5 function.

A well-characterized function of MDA5 is the sensing of viral RNA and thereafter activation of down-stream signalling events leading to the expression of type I and III IFNs^3^,^4^,^36^. MDA5 is expressed by human islets (Fig. 2d 26) and contributes to the host immune response to CVBs (e.g.^27^,^28^). Here, we observed significantly higher expression levels of both IFNλ1 and IFNλ2 mRNA in CVB3 infected islets carrying the TC genotype compared to the TT genotype (p < 0.05; Fig. 3b). This is a novel and previously undiscovered correlation between rs1990760 and the magnitude of type III IFN expression in response to a relevant human pathogen. Because we noted a difference in type III and not type I IFN expression (Fig. 3a), these observations also indicate that the impact of the rs1990760 polymorphism may be cell type and/or tissue dependent, and highlight the importance of studying disease relevant cell types when assessing the impact of disease-associated SNPs (e.g.^15^).

A previous study uncovered differences in the intracellular events leading to IFNβ and IFNλ production^3^; while the transcription of both IFN families is induced through the activation of the RIG-I like PRRs (RIG-I and MDA5) via the adaptor protein MAVS, the organelle association of MAVS determines which types of IFNs are transcriptionally induced following microbial stimulation. MAVS associated with mitochondria has the ability to induce both type I and III IFNs, while MAVS associated with peroxisomes selectively induces type III IFN production following viral infection in an IRF-1 dependent fashion^3^. Given that our study discovered a distinct rs1990760 genotype-specific IFNλ expression we speculate that in infected human islet cells MDA5 might primarily act through peroxisomal MAVS to induce IFNλ expression. IFNβ mRNA expression showed no such correlation to the rs1990760 TT and TC genotypes (Fig. 3), suggesting that type I IFNs might be induced via other PRR(s) (Fig. 6).

Recent studies have shown that the transcription factor IRF-1 specifically regulates the expression of type III IFNs (and not type I IFNs) in response to infection with RNA viruses (reviewed in ref. 3). Here, we found that the expression of IRF-1 was significantly induced in CVB3 infected islets from donors with the TC genotype but not in islets from donors carrying the TT genotype (p < 0.01; Fig. 4). We also discovered a correlation between the gene expression levels of IRF-1 with those of IFNλ1 or IFNλ2 in islets with the TC genotype but not the TT genotype (Fig. 5). Based on this we hypothesize that varying levels of IRF-1 expression contribute to the differences in type III IFN expression observed between the two genotypes (Fig. 6).

IRF-1 is an IFN-inducible gene and its expression is induced in human pancreatic islets by IFN-treatment^41^. It is therefore possible that IRF-1 contributes to type III IFN production in CVB3 infected islets via a positive feedback loop, and that the overall stronger type III IFN response in islets with the TC genotype is a result of a stronger positive feedback on type III IFN gene expression. The transcription factor BATF2 was recently shown to interact with IRF-1 and to positively influence the transcription of a subgroup of genes in IFNγ stimulated macrophages^42^. Our transcriptome analysis suggested that CVB3 infected islets with the TC genotype express more BATF2 mRNA than islets with the TT genotype (Fig. 4). Thus, it is possible that BATF2 interacts with IRF-1 and that this further augments type III IFN gene expression. Future studies are needed to assess the role for BATF2 in regulating IFN gene transcription.

Our transcriptome analyses and functional annotations showed that human islets respond to CVB3 infection by upregulating numerous ISGs (often referred to as the type I IFN signature, Fig. 1). The expression pattern resembled that previously observed in human pancreatic islets stimulated with type I or type III IFNs^26^,^29^, further supporting a key role for IFNs in inducing gene transcription following CVB3 infection. We also discovered that islets carrying the TC genotype respond to CVB3 infection with a stronger ISG gene expression than islets carrying the TT genotype. This difference was not limited to gene expression, but was also evident on the protein level as demonstrated by differences in secreted CXCL10 (Fig. 3h). A more substantial IFN signature in the islets from TC donors lend further weight to the hypothesis that donors carrying this genotype produce more IFNs following infection compared to TT donors. In addition to this, our results also suggest that in this setting, donors carrying the T1D risk genotype (TT) have a weaker response to CVB3 infection than donors carrying the protective TC genotype. In support for this conclusion, we recently observed that peripheral blood mononuclear cells (PBMCs) from donors carrying the rs1990760 TC genotype respond with a higher ISG expression upon stimulation with CVB-antibody complexes as compared to PBMCs from TT carriers^43^.

As suggested by virus titrations and PCR measurements, TT and TC genotypes do not differ dramatically in their ability to control Coxsackievirus replication in pancreatic islets (Fig. 3i). Still, there was a trend towards higher expression of CVB3 RNA in islets from donors carrying the TC genotype as compared to donors carrying the TT genotype (p = 0.09). Interestingly, Cinek *et al*. measured the presence of enterovirus RNA in peripheral blood samples from healthy children genotyped for the rs1990760 polymorphism and reported a higher frequency of enterovirus RNA in individuals with the heterozygous genotype (TC, 14.4%) compared to individuals with the TT (9.5%) or CC (7%) genotypes^44^. Taken together, this and our study suggest that there may be subtle, yet important biological differences between the genotypes in terms of controlling enterovirus replication. This is in line with other investigations indicating a weak but possible link between the rs1990760 polymorphism in the clearance of both enterovirus 71^44^,^45^ and hepatitis C virus^16^,^30^.

Based on the observations we made in this study, we propose that IRF-1 contributes to CVB3 induced type III IFN expression via a positive feedback loop. We also suggest that the more potent type III IFN response in islets with the TC genotype is a result of an overall stronger positive feedback on type III IFN gene expression, and that this leads to a more robust ISG response. The mechanism behind the stronger positive feedback in donors with the TC genotype remains speculative. We however suggest that subtle differences in virus replication, with slightly higher levels of virus replication in TC donors, results in a stronger activation of MDA5 in islets from donors with this genotype. Via positive feedback mechanisms, including the expression of genes coding for transcription factors important for the expression of type III IFNs, this somewhat greater response unfolds in a statistically significant stronger type III IFNs and ISG response in TC donors as compared to TT donors.

In summary, our study provides important insights into how the host immune response to a clinically relevant virus may differ depending on the rs1990760 (A946T) genotype. Our studies also put an emphasis on the fairly newly described group of type III IFNs. The link between the strength of their expression and the rs199760 polymorphism in IFIH1 warrants further investigations on the role of type III IFNs in infectious and autoimmune diseases.

## Methods

### Human pancreatic islets

Human pancreatic islets were isolated by the Nordic Network for Islet Transplantation in Uppsala, Sweden, as previously described^46^. All pancreas donors came from the Nordic countries Sweden, Norway or Finland (further information regarding donor ethnicity was not provided). Human islets from 29 donors (12 females and 17 males. Islets from 6 donors were used for Western blot analyses and islets from the other 23 donors for gene expression studies) were isolated from human cadavers, with the average donor age being 59 ± 11 years (range 25 to 78 years) and the cold ischemia time 10 ± 4 h (range 4.3 to 21.4 h). The quality of the islets was evaluated by insulin release in response to glucose, and stimulation index for the donors were calculated as 10.5 ± 7.4 (range 2.2 to 31.2) by using a dynamic perfusion system previously described^46^. Stimulation indexes were available for all donors except one. The islet preparations had a purity of 69 ± 19% (range 27–99%), determined by dithizone staining, and were before the experiments started further purified by hand picking. Islets were initially maintained in CMRL-1066 supplemented with 2 mM L-glutamine, 10% inactivated human serum, 10 mM HEPES, 0.25 μg/ml Fungizone, 505 μg/ml Gentamycin, 105 μg/ml ciprofloxacin and 10 mM nicotinamide. Upon arrival at Karolinska Institutet, islets were transferred to RPMI 1640 supplemented as above but with inactivated fetal bovine serum instead of human serum and without nicotinamide. Human islets were transported to Karolinska Institutet 1–9 days after isolation and experiments were performed after 2–13 days of recovery. Organ donors or their relatives provided their informed consent to donate islets. The experimental protocols and experiments were approved by local ethic committees in Uppsala (Forskningsetikkomittén, Uppsala University) and Stockholm (Forskningsetikkomitté Syd), and performed in accordance with the principles of the Declaration of Helsinki 2000 and the Department of Health and Human Services Belmont Report.

### Virus stock, virus titration and infection

CVB3 Nancy was propagated and the titer was determined using HeLa cells. Human islets were infected for 90 minutes with CVB3 Nancy at 4 × 10^4^ PFU/islet in 3 ml serum free media. Each experiment comprised of control (mock-infected) and virus infected islets from the same donor. After infection cells were washed three times using complete media, placed in 3 ml media and incubated at 37 °C for 24 h or 48 h. At indicated time points, supernatants were stored at −80 °C and the islets were lysed using RLT buffer (Qiagen) and stored at −80 °C. Viral titer in supernatant and in the last wash supernatant was determined by standard plaque assay using HeLa cells. Viral titers were determined as PFU/ml and presented as Log_10_ PFU/ml. The detection limit of the assay was 2.5 PFU/ml.

### DNA isolation and SNP genotyping

Total DNA was purified from whole blood or human islets using DNeasy Blood and Tissue kit (Qiagen) or Allprep RNA/DNA/Protein kit (Qiagen). Quantity and purity of DNA was measured using a NanoDrop ND-1000 (Saveen and Werner AB, Sweden). Genotyping of each donor was performed using TaqMan SNP Genotyping Assay (Applied Biosystem) and custom made probes detecting the SNPs rs1990760 and r3747517 in IFIH1 according to manufacturer’s instructions. The genotype was automatically determined by measuring the allele specific fluorescence using an ABI Prism 7500 Sequence Detection System (SDS) and the SDS software v2.0.3 program for allelic discrimination (Applied Biosystems).

### RNA isolation, cDNA synthesis and quantitative real-time PCR

Total RNA (>200 bp) was isolated using RNeasy kit or Allprep RNA/DNA/Protein kit (Qiagen) according to manufacturer’s instructions. Quantity and purity of RNA was assessed using a NanoDrop ND-1000 (Saveen and Werner AB, Sweden). Total RNA (0.27 μg islet cell RNA or 1 μg MEFs RNA) was treated with DNase I or Turbo DNase (Life Technologies, Sweden) and converted into cDNA using random hexamers and SuperScript III First Strand Synthesis System for RT-PCR (Invitrogen, Sweden). Genomic DNA contamination of the cDNA was assessed in each sample. Quantitative real-time PCR was performed using an ABI Prism 7500 Sequence detecting system. Quantification of human IFNβ, MDA5, RIG-I, TLR3, MxA, IRF-1 and GAPDH mRNA was performed using QuantiTect Primer Assay (Qiagen). CVB was detected using specific primers (forward: 5-GGCCCCTGAATGCGGCTA AT-3′, reverse: 5′-CAATTGTCACCATAAGCAGCC-3′) (Invitrogen) and RT^2^ Real-time^TM^ SYBR green/ROX PCR master mix (SuperArray, Sweden). Quantification of the human gene transcripts IFNβ, IFNλ1, IFNλ2, CXCL10 and GAPDH was performed using specific TaqMan probes (Applied Biosystem) and TaqMan mastermix (Applied Biosystem). The mRNA expression of each gene was run in triplicate and normalized to the expression levels of GAPDH. Data is presented as relative expression compared to GAPDH (2^−(Ct gene of interest− Ct GAPDH)^ or 2^−(ΔCt)^) or as fold induction (2^−(ΔCt infected−ΔCt mock-infected)^ or 2^−(ΔΔCt)^) or as percent of an indicated sample. Note that due to the lack of basal mRNA expression of IFNs, the inducible expression of IFN genes was not shown as a “fold-induction”, but rather as 2^−ΔCt^ values.

### RNA sequencing

For RNA sequencing, libraries were prepared from 100 ng of total RNA samples (all with good quality; RIN values of >8.0 when analysed with Agilent 2100 Bioanalyzer) using Illumina TruSeq^®^ RNA Sample Preparation kit v2 (Catalog ID: RS-122–2001) and following Illumina’s guidelines (part # 15026495). Briefly, polyA+ RNA was purified, fragmented, converted to cDNA and end-repaired followed by adaptor ligation and sample indexing using Unique Illumina TruSeq indexing adapters. The products were then purified and enriched to create the final cDNA library. Libraries were quantified with Qubit^®^ Fluorometric Quantitation (ThermoFischer Scientific) and quality was assured with Agilent Bioanalyzer 2100. All libraries showed excellent quality, with a fragment size in the range of 200–700 bp (with an average of 250–350 bp). The clonal cluster amplification was carried out with Illumina cBOT System. The amplified libraries were pooled and sequenced with Illumina HiSeq2500 Next-Generation Sequencing platform (using TruSeq v3 sequencing chemistry) in two lanes of a paired-end high output run with read length of 100 bp. The run generated a total of 250 × 10^6^ and 255 × 10^6^ raw reads passing filter with Q30% score of 88.9% and 89.3%, for each of the two lanes, respectively.

### RNAseq data processing and analysis

The quality control of raw sequencing reads was performed with FastQC, (www.bioinformatics.babraham.ac.uk/projects/fastqc/), and adapters and low quality bases were trimmed by Trimmomatic^47^. The trimmed reads were then aligned to the human reference genome GRCh37.75 (Ensembl release 75) using Tophat2^48^. Htseq-count^49^ summarized read counts for each gene. The R/Bioconductor packages edgeR^50^ and limma^51^ were used to identify differentially expressed genes. To detect genes that react significantly differently to the infection between TT and TC groups, separate differential expression (DE) analyses within the TT and TC groups (specifically, in each group, we performed a paired DE test between samples before infection and after infection) were made. Genes with a false discovery rate (FDR) < 0.05 in either comparison were considered significant. Among these genes, the subset was identified, which were also differentially expressed between the TT and TC groups (FDR < 0.05 and absolute fold change >2). Hierarchical clustering was performed using the Pearson correlation between log_2_(CPM) values as the distance measure, and average linkage. Data enrichment analysis was performed using the MetaCore^TM^ (Thosmon Reuters) analysis tool. Results on non-coding RNAs were excluded from this study and will be separately reported in a separate manuscript.

### Antibodies, SDS-PAGE and Western blot

Western blot was performed as described before^29^. Primary antibodies were added and incubations were done over night at 4 °C: MDA5 (1:1000, Alexis Biochemicals) RIG-I (1:1000, Alexis Biochemicals), and antibody detection was done with HRP conjugated anti-rabbit antibody (1:1000, Bio-Rad, Sweden). To guarantee equal loading, membranes were probed with a primary antibody against actin (1:30.000, MP Biomedicals, Aurora, Ohio, USA), followed by incubation with HRP conjugated anti-mouse antibody (1:10.000, Bio-Rad, Sweden).

### ELISA

Secreted CXCL10 was measured in islet culture supernatants using the BD OptEIA™ human CXL10 ELISA kit (BD Biosciences) according to manufacturer’s instructions. The capture antibody was immobilized on maxisorp ELISA plates (Thermo Scientific) using the BD OptEIA™ reagent set B (BD Biosciences). Optical density (OD) was measured at 450 nm with a correction at 570 nm using the xMark Microplate spectrophotometer (Bio-Rad). The detection limit of the assay was 7 pg/ml. The results were normalized to the RNA content of each sample and given as ng/ng isolated islet RNA. RNA content was used since the islets had been harvested for RNA extraction and data for protein and DNA content was therefore not available.

### Statistical analysis

Statistical analysis was performed using GraphPad Prism 5. A normality test (Kolmogorov-Smirnov test) was used to determine whether the data sets were normally distributed. Multiple comparison was performed by one way Anova. When comparing two groups Wilcoxon matched-pairs signed rank test (paired samples) or Mann-Whitney test (unpaired samples) was used. Correlations were performed using Pearson correlation test. Data is presented as mean ± S.E.M.

### URLs

Database of IFN regulated genes, Interferome, http://www.interferome.org; web based resource of genetics and genomics of T1D susceptibility, T1DBase, http://www.t1dbase.org; MetaCore^TM^ enrichment analysis tool, https://www.portal.genego.com/.

## Additional Information

**How to cite this article**: Domsgen, E. *et al*. An IFIH1 gene polymorphism associated with risk for autoimmunity regulates canonical antiviral defence pathways in Coxsackievirus infected human pancreatic islets. *Sci. Rep.* **6**, 39378; doi: 10.1038/srep39378 (2016).

**Publisher's note:** Springer Nature remains neutral with regard to jurisdictional claims in published maps and institutional affiliations.

## Supplementary Material

Supplementary Tables and Figures
