# Supplementary material for: An IFIH1 gene polymorphism associated with risk for autoimmunity regulates canonical antiviral defence pathways in Coxsackievirus infected human pancreatic islets
Source: Sci Rep. 2016 Dec 21;6:39378. doi: 10.1038/srep39378 (PMC5175199; doi:10.1038/srep39378)
Supplement: Supplementary Tables and Figures [file srep39378-s1.pdf]

SUPPLEMENTARY FIGURES AND FIGURE LEGENDS

An IFIH1 gene polymorphism associated with risk for autoimmunity regulates canonical antiviral defense pathways in Coxsackievirus infected human pancreatic islets

Erna Domsgen[1,†], Katharina Lind[1,†], Lingjia Kong[2], Michael H. Hühn[1,#], Omid Rasool[2], Frank van Kuppeveld[3], Olle Korsgren[4], Riitta Lahesmaa[2], Malin Flodström-Tullberg[1,5,*]

Supplementary Table S1: Frequencies of rs1990760 and rs3747517 genotypes in the human islet donor cohort.

SNP combination		rs3747517, number of donors (%)		
		TT 843H/843H	TC 843H/843R	CC 843R/843R
rs1990760 genotype, number of donors (%)	TT 946T/946T	0 (0%)	1 (4.3%)	9 (39.1%)
	TC 946T/946A	1 (4.3%)	10 (43.5%)	2 (8.7%)
	CC 946A/946A	0 (0%)	0 (0%)	0 (0%)

Total DNA was purified from all donors (n=23). Genotyping was performed using TaqMan SNP Genotyping Assay (Applied Biosystem) and custom made probes detecting the SNPs rs1990760 and re3747517 in *IFIH1* according to manufacturer's instructions.

Supplementary Table S2: Genes with a differentially expression in CVB3 infected as compared to uninfected human pancreatic islets, identified by RNA Seq analysis.

Gene ID	logFC	ave expr	p-value	adj. p-value	ISG
ANGPTL1	1.6680	5.4121	< 0.001	0.004	
ANO7P1	1.1182	2.6015	0.002	0.027	
APOBEC3G	1.3749	5.9196	< 0.001	0.003	*
APOL2	1.0204	8.6426	< 0.001	< 0.001	*
APOL3	1.9312	5.0744	< 0.001	0.003	*
APOL6	1.1045	9.5803	< 0.001	< 0.001	*
ATF3	1.1785	8.2002	< 0.001	< 0.001	*
ATP10A	1.8773	4.4964	< 0.001	0.004	*
BATF2	2.9283	6.0873	< 0.001	< 0.001	*
BRCA2	1.2109	2.2653	0.002	0.032	*
BST2	3.0557	7.1839	< 0.001	< 0.001	*
C19orf66	1.3095	7.1936	< 0.001	< 0.001	*
C3AR1	1.3080	3.6546	< 0.001	0.014	
CCL3	3.1280	2.7892	< 0.001	0.002	
CCL4	4.4167	2.8681	< 0.001	< 0.001	*
CCL5	4.8863	5.1560	< 0.001	0.003	*
CCL7	3.0849	2.7217	< 0.001	0.007	*
CCL8	5.4265	2.5597	< 0.001	< 0.001	*
CCR1	1.4342	4.2996	< 0.001	0.007	*
CCR5	1.4459	2.7961	0.005	0.048	
CD274	2.2761	5.8411	< 0.001	< 0.001	
CD38	4.1035	2.5713	< 0.001	0.005	*
CD40	1.0381	6.7912	0.002	0.032	*
CD48	1.6154	2.0165	0.004	0.043	
CDON	1.1147	6.4416	0.002	0.025	
CMPK2	3.1804	7.8305	< 0.001	< 0.001	*
CSF2	2.3567	3.3119	0.003	0.036	*
CSF2RB	1.2053	2.7438	0.004	0.041	*
CX3CL1	2.6423	8.9196	< 0.001	< 0.001	*
CXCL10	7.2068	7.0234	< 0.001	< 0.001	*
CXCL11	6.8187	6.0957	< 0.001	< 0.001	*
CXCL9	2.9833	5.0685	0.003	0.041	*
CYP2J2	1.6249	5.3773	< 0.001	0.011	*
DAPP1	1.7108	4.7216	0.003	0.038	*
DDX58	3.1140	8.4626	< 0.001	< 0.001	*
DDX60	2.6847	8.3295	< 0.001	< 0.001	*
DDX60L	2.1386	7.6171	< 0.001	< 0.001	*
DHX58	2.4373	6.2969	< 0.001	< 0.001	*
DTX3L	1.1845	9.4227	< 0.001	< 0.001	*
EIF2AK2	1.5267	9.1297	< 0.001	< 0.001	*
EPSTI1	2.1299	7.9304	< 0.001	< 0.001	*
ETV7	2.1507	5.8934	< 0.001	< 0.001	*
FAM26F	1.6409	2.1275	0.002	0.025	*
FBXO6	1.0612	6.7463	< 0.001	0.003	*
FPR3	1.2792	4.2031	0.005	0.046	*
GBP1	3.1496	7.4023	< 0.001	< 0.001	*
GBP4	3.8405	6.4380	< 0.001	< 0.001	*
GBP5	4.9161	2.9652	< 0.001	0.002	*
GJA4	2.0068	2.6018	0.002	0.025	

Gene ID	logFC	ave expr	p-value	adj. p-value	ISG
GMPR	1.9156	6.5507	< 0.001	< 0.001	*
GPR123	1.1373	3.6004	< 0.001	0.018	
HELZ2	2.4625	8.8511	< 0.001	< 0.001	*
HERC5	2.8024	7.7210	< 0.001	< 0.001	*
HERC6	2.2861	8.1665	< 0.001	< 0.001	*
HES4	1.2929	5.8300	0.002	0.028	*
HIST1H3D	2.8016	0.2008	< 0.001	0.017	
HSH2D	2.4424	6.0673	< 0.001	< 0.001	*
IDO1	4.0871	5.6550	< 0.001	0.004	*
IFI16	1.5503	7.9529	< 0.001	0.008	*
IFI27	3.9139	6.6092	< 0.001	< 0.001	*
IFI35	1.9853	7.9441	< 0.001	< 0.001	*
IFI44	3.5327	6.9914	< 0.001	< 0.001	*
IFI44L	4.8202	6.0911	< 0.001	< 0.001	*
IFI6	2.8065	10.5049	< 0.001	< 0.001	*
IFIH1	2.1495	8.7764	< 0.001	< 0.001	*
IFIT1	4.5611	9.2418	< 0.001	< 0.001	*
IFIT2	4.4304	9.0720	< 0.001	< 0.001	*
IFIT3	4.1091	9.6976	< 0.001	< 0.001	*
IFIT5	1.1341	8.1653	< 0.001	< 0.001	*
IFITM1	3.6231	7.4472	< 0.001	< 0.001	*
IFNB1	6.1882	-0.7817	< 0.001	0.008	
IFNL1	7.8416	0.3314	< 0.001	< 0.001	
IFNL2	7.5010	-0.0363	< 0.001	< 0.001	
IFNL3	7.0969	-0.3253	< 0.001	< 0.001	
IL12A	2.2062	1.4745	< 0.001	0.017	
IL18BP	1.2119	6.8220	< 0.001	0.016	*
IL18R1	1.2423	4.1564	< 0.001	0.006	
IL1RN	1.7166	6.7621	< 0.001	0.007	*
IL4I1	2.2930	4.8346	< 0.001	< 0.001	
IRF1	1.3070	8.8445	< 0.001	0.003	*
IRF7	2.7038	7.1657	< 0.001	< 0.001	*
ISG15	4.6685	8.7913	< 0.001	< 0.001	*
ISG20	2.2582	7.2391	< 0.001	< 0.001	*
KLHDC7B	2.4087	4.0534	0.002	0.026	
KLK10	2.3866	2.8510	0.004	0.041	
KRT6B	2.3302	5.2296	0.002	0.029	
LAMP3	4.1727	6.7808	< 0.001	< 0.001	*
LAP3	1.9876	8.7632	< 0.001	< 0.001	*
LY6E	2.0493	9.5391	< 0.001	0.002	*
MAP3K8	1.1732	5.8886	0.004	0.041	
MLKL	2.0919	5.4017	< 0.001	< 0.001	*
MNDA	3.2217	0.5666	< 0.001	0.015	
MT2A	1.0865	8.9259	0.003	0.038	*
MX1	3.8984	9.7365	< 0.001	< 0.001	*
MX2	5.0793	6.5469	< 0.001	< 0.001	*
NFKBIA	1.3580	9.3462	0.001	0.025	
NLRC5	1.4753	7.0624	< 0.001	0.002	*
NMI	1.3269	7.0775	< 0.001	0.003	*
NOD2	2.8120	1.5483	< 0.001	0.017	*
NR1D1	1.2792	8.1482	< 0.001	0.011	
NT5C3A	1.2672	8.3105	< 0.001	0.010	*
NUAK2	1.0454	7.3051	0.005	0.047	

Gene ID	logFC	ave expr	p-value	adj. p-value	ISG
OAS1	3.8306	8.5330	< 0.001	< 0.001	*
OAS2	5.3518	6.7840	< 0.001	< 0.001	*
OAS3	3.8714	8.9674	< 0.001	< 0.001	*
OASL	5.3674	6.7185	< 0.001	< 0.001	*
PARP10	1.5736	8.1975	< 0.001	< 0.001	*
PARP12	1.6433	8.2346	< 0.001	< 0.001	*
PARP14	1.8360	9.1803	< 0.001	< 0.001	*
PARP9	1.3593	9.2668	< 0.001	< 0.001	*
PLEK	1.3541	3.1828	0.003	0.039	*
PLEKHA4	1.5398	7.2098	< 0.001	0.004	*
PLSCR1	1.4048	9.3150	< 0.001	0.008	*
PMAIP1	1.2867	6.6753	0.002	0.026	*
PML	1.4283	8.3345	< 0.001	< 0.001	*
PNPT1	1.6906	7.6710	< 0.001	< 0.001	*
PSMB9	1.7392	7.2025	< 0.001	< 0.001	*
RARRES3	1.0809	6.6594	0.004	0.041	*
RBM11	1.1071	4.0179	< 0.001	0.017	
RHEBL1	1.5204	0.9154	0.003	0.038	
RSAD2	3.7239	9.3255	< 0.001	< 0.001	*
RTP4	1.6606	6.1432	< 0.001	0.001	*
SAMD9	2.5657	8.1743	< 0.001	< 0.001	*
SAMD9L	3.1415	6.9706	< 0.001	< 0.001	*
SAMHD1	1.9739	8.1743	< 0.001	< 0.001	*
SECTM1	2.1212	6.4486	< 0.001	0.006	*
SERPINB9	1.0854	6.6196	0.002	0.027	*
SIGLEC1	4.1149	1.8549	< 0.001	< 0.001	*
SLC15A3	2.2887	5.2661	< 0.001	< 0.001	*
SLC25A28	1.3594	6.7804	< 0.001	< 0.001	*
SORL1	1.1413	9.5385	0.002	0.026	*
SP100	1.3264	8.7940	< 0.001	0.004	*
SP110	2.3446	6.6723	< 0.001	< 0.001	*
STARD5	1.6809	3.5776	< 0.001	0.006	*
STAT1	2.0124	10.6262	< 0.001	< 0.001	*
STAT2	1.1693	8.6411	< 0.001	0.003	*
STX11	1.1859	3.8796	0.004	0.041	*
SUSD3	2.1988	1.0891	0.001	0.019	
TAGAP	1.9399	2.3619	< 0.001	< 0.001	*
TAP1	1.7751	9.2956	< 0.001	< 0.001	*
TAP2	1.6983	8.4546	< 0.001	< 0.001	*
TDRD7	1.1289	7.7630	< 0.001	< 0.001	*
THEMIS2	1.1482	6.4367	< 0.001	0.010	*
TLDC2	1.3456	1.6951	0.003	0.038	
TMEM140	1.3979	7.7030	< 0.001	0.002	*
TNF	2.2476	3.2585	0.002	0.033	*
TNFSF10	1.1955	10.1610	0.003	0.038	*
TNFSF13B	2.9497	4.8577	< 0.001	< 0.001	
TRANK1	1.4338	6.5278	< 0.001	0.013	*
TREX1	1.4506	6.3311	< 0.001	< 0.001	*
TRIM14	1.6150	7.5359	< 0.001	< 0.001	*
TRIM21	1.3428	7.3726	< 0.001	0.003	*
TRIM22	1.9970	7.9421	< 0.001	0.003	*
TRIM25	1.3168	8.8707	< 0.001	< 0.001	*
TXNIP	1.1586	9.7417	0.004	0.044	*

Gene ID	logFC	ave expr	p-value	adj. p-value	ISG
TYMP	1.4427	8.4857	0.004	0.043	*
UBA7	1.2606	6.9539	0.002	0.028	*
UBE2L6	1.6487	8.8429	< 0.001	< 0.001	*
UNC93B1	1.3504	7.8263	< 0.001	< 0.001	*
USP18	2.7723	7.2247	< 0.001	< 0.001	*
WARS	1.2006	9.7545	< 0.001	0.003	*
XAF1	4.0922	5.0292	< 0.001	< 0.001	*
ZBP1	5.5505	1.5168	< 0.001	< 0.001	*
ZC3HAV1	1.1179	8.5393	< 0.001	0.001	*
ZNF433	1.1365	4.6235	0.004	0.041	
ZNFX1	1.4038	9.1643	< 0.001	< 0.001	*

The logFC and the average expression (ave. expr.) for control vs. CVB3 infected islets. Genes identified as interferon stimulated genes (ISGs) by the Interferome database are marked with (*). logFC, log_2 fold change (control vs CVB3 infected human islets); ave expr, average log_2 expression of the gene across all samples; adj. p-value, adjusted p-value; ISG, Interferon stimulated gene.

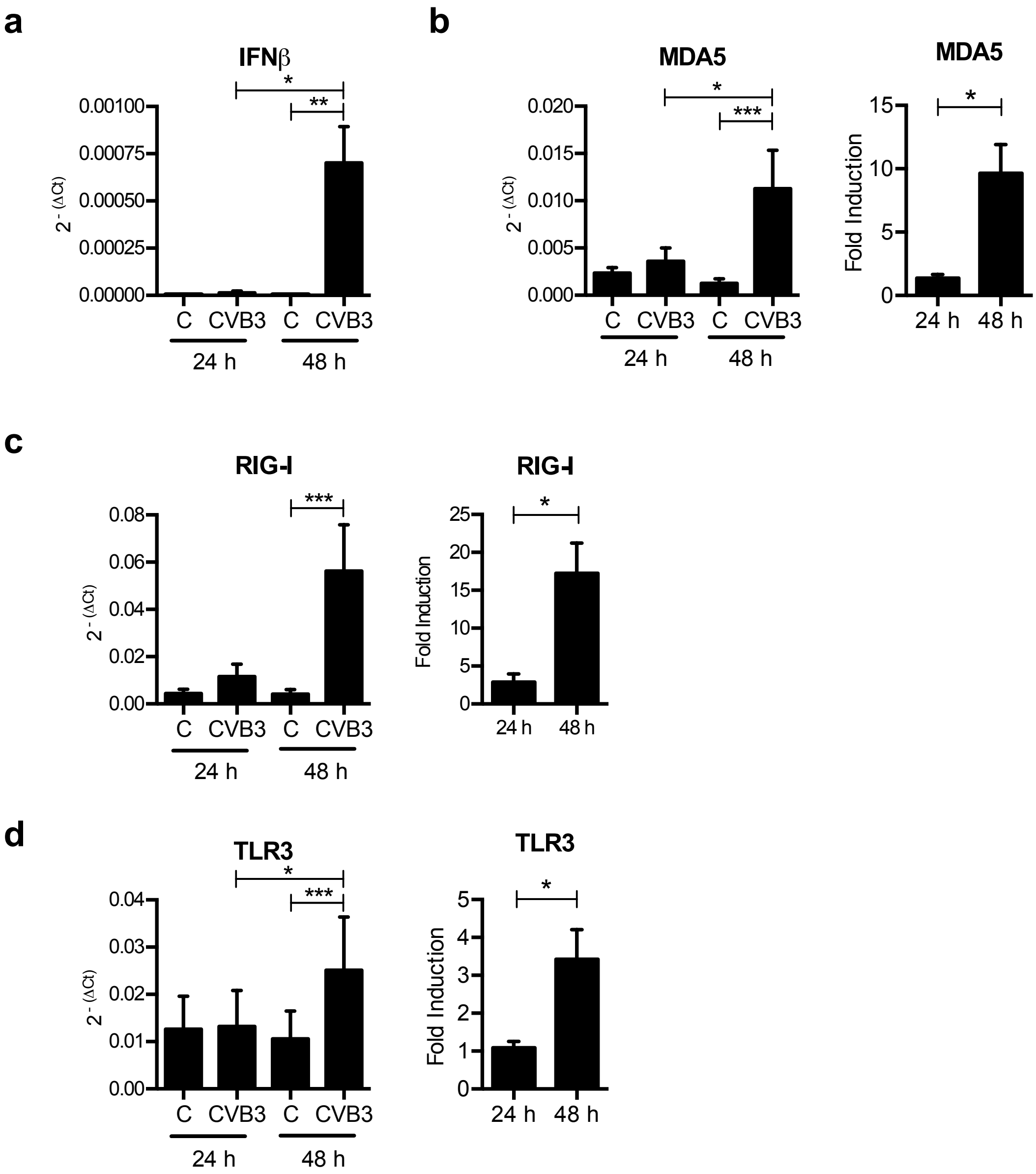

Supplementary Fig. S1. Human pancreatic islets respond to CVB3 infection by upregulating the expression of IFNβ and PRRs.

Human islets from seven donors were infected with CVB3. At 24 h and 48 h p.i. total mRNA was isolated and the expression levels of IFNβ (A), MDA5 (B), RIG-I (C) and TLR3 (D) were determined using quantitative real-time (RT)-PCR. The expression levels were normalized to GAPDH and presented as $2^{-(\Delta Ct)}$, or normalized to GAPDH and compared to uninfected control from the same donor and presented as $2^{-(\Delta\Delta Ct)}$. Data is shown as mean ± SEM, * $p<0.05$, ** $p<0.01$, *** $p<0.001$, Friedmann test with Dunns correction or Wilcoxons matched-paired signed test.

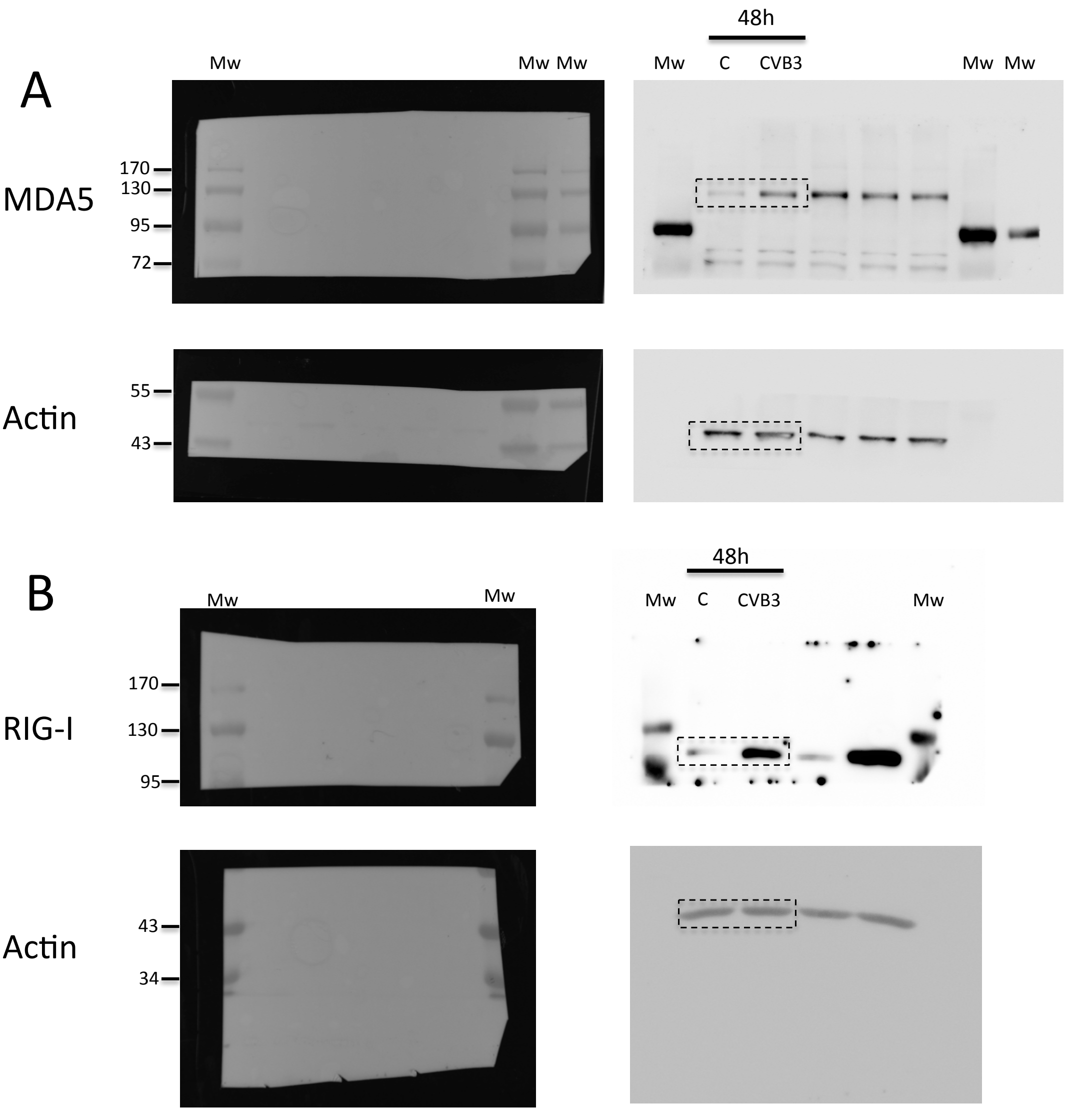

Supplementary Fig. S2: Uncropped full-length images of Western blot membranes presented in the main Figure 2d.

As indicated, the membranes were cut to enable simultaneous blotting for two antibodies MDA5 (A) or RIG-I (B) and actin without the need for membrane stripping.

T1D associated genes

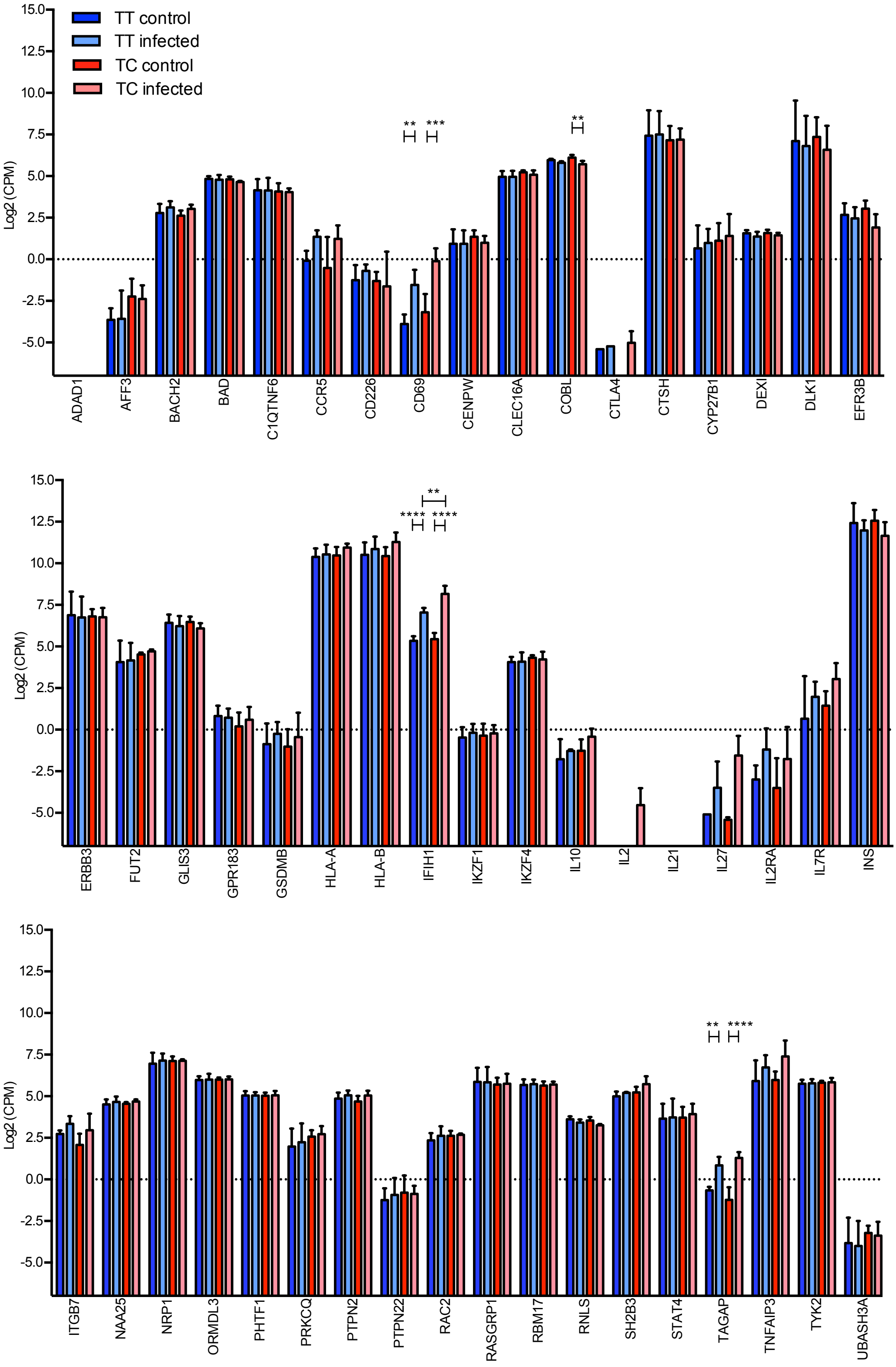

Supplementary Fig. S3: Four T1D-associated genes (CD69, COLB, IFIH1, TAGAP) are differentially expressed in pancreatic human islets upon CVB3 infection.

RNA sequencing was performed on RNA isolated from non-infected and CVB3 infected primary human islets 48 h p.i. The mRNA abundance of genes associated with type 1 diabetes (except of HLA II genes, based on www.T1Dbase.org, analysis made on May 23rd, 2016) is shown as $\log_2$(CPM) in control and CVB3 infected islets carrying the rs1990760 TT (TT control, TT infected) and TC genotype (TC control, TC infected). Data is shown as mean ± SEM, ** $p<0.01$, *** $p<0.001$, **** $p<0.0001$, one-way ANOVA with Bonferroni correction.
